# Reference Intervals for N-Terminal Pro-B-Type Natriuretic Peptide in Amniotic Fluid between 10 and 34 Weeks of Gestation

**DOI:** 10.1371/journal.pone.0114416

**Published:** 2014-12-09

**Authors:** Waltraut M. Merz, Christina Leufgen, Rolf Fimmers, Birgit Stoffel-Wagner, Ulrich Gembruch

**Affiliations:** 1 Department of Obstetrics and Prenatal Medicine, University Bonn Medical School, Bonn, Germany; 2 Institute for Medical Biometry, Informatics and Epidemiology, University Bonn Medical School, Bonn, Germany; 3 Institute for Clinical Chemistry and Pharmacology, University Bonn Medical School, Bonn, Germany; Virgen Macarena University Hospital, School of Medicine, University of Seville, Spain

## Abstract

**Background:**

In adult and pediatric cardiology, n-terminal pro-B-type natriuretic peptide (nt-proBNP) serves as biomarker in the diagnosis and management of cardiovascular dysfunction. Elevated levels of circulating nt-proBNP are present in fetal conditions associated with myocardial pressure or volume load. Compared to fetal blood sampling, amniocentesis is technically easier and can be performed from early pregnancy onwards. We aimed to investigate amniotic fluid (AF) nt-proBNP concentrations in normal pregnancies between 10 and 34 weeks of gestation.

**Methods:**

Nt-proBNP and total protein (TP) was measured in AF by chemiluminescence assay (photometry, respectively). To adjust for a potential dilutional effect, the AF-nt-proBNP/AF-TP ratio was analyzed. Reference intervals were constructed by regression modeling across gestational age.

**Results:**

132 samples were analyzed. A negative correlation between AF-nt-proBNP/AF-TP ratio and gestational age was observed. Curves for the mean and the 5% and 95% reference interval between 10 and 34 weeks of gestation were established.

**Conclusion:**

In normal pregnancy, nt-proBNP is present in AF and decreases during gestation. Our data provide the basis for research on AF-nt-proBNP as biomarker in fetal medicine.

## Introduction

In adult and pediatric patients with heart failure, analysis of brain natriuretic peptide (BNP) or its inactive cleavage product, n-terminal pro-B-type natriuretic peptide (nt-proBNP) allows a comprehensive assessment of the myocardial structure, function and loading. Nt-proBNP serves as an established biomarker in the diagnosis and management of myocardial dysfunction, and nt-proBNP-guided heart failure therapy has been incorporated into clinical practice [1]–[5]. BNP is secreted from cardiac myocytes in response to stretch; together with atrial natriuretic factor (ANF) it regulates pre- and afterload by increasing natriuresis and antagonizing the renin-angiotensin-aldosteron system. BNP is additionally assigned a central role in cardiac remodeling, knock-out mice develop myocardial fibrosis [6].

A variety of fetal conditions are associated with myocardial dysfunction. These include structural cardiac defects, primary myocardial diseases and cardiomyopathies; diseases which imply an increase in cardiac output or altered intrathoracic volume relations (anemia, arterio-venous malformations, sacrococcygeal teratoma, cystic-adenomatoid pulmonary malformation); twin-twin-transfusion syndrome (TTTS) in monochorionic pregnancies; and fetal growth restriction. Progress in minimally-invasive surgical techniques allows intrauterine therapy for some of the aforementioned conditions; close surveillance and timely delivery is applied to the others [7]–[9]. Assessment of fetal myocardial function is undertaken by echocardiography, Doppler sonography of the fetal vasculature, myocardial tissue Doppler, and speckle tracking. Limitations of these image-based methods include the influence of cardiac loading conditions and the inter- and intraobserver variability [10], [11]. Recently, nt-proBNP was investigated for its potential role as biomarker during fetal life. Four- to tenfold elevated levels of circulating nt-proBNP were found in anemia, cardiac and urinary tract malformations and fetal growth restriction, conditions associated with increased pressure or volume load. Nt-proBNP analysis provided evidence of changes in myocardial characteristics and cardiac remodeling [12]–[20].

The incorporation of nt-proBNP into clinical practice is hampered by the invasiveness of fetal blood sampling (FBS) and its limitation to late stages of pregnancy. Unlike FBS, amniocentesis (AC) is characterized by a high success and low complication rate, and can be performed from 14 weeks of gestation onwards. Furthermore, access to the amniotic cavity is part of any intrauterine intervention.

The aim of our study was to analyze nt-proBNP in amniotic fluid (AF) and establish reference intervals as a basis for investigations into its suitability as biomarker in fetal medicine.

## Patients and Methods

### 2.1. Study population

The study was approved by the University Bonn Medical School Ethics Committee (registration number 305/11). All participating women gave their written consent. Patients with gestational age (GA) between 10 and 34 weeks, presenting at our Center for Prenatal Diagnosis between November 2011 and August 2013 who were scheduled to undergo any invasive intervention that implies access to the amniotic cavity were eligible. No procedure was undertaken for the sole purpose of the study.

A detailed assessment of the fetal anatomy and cardiovascular status including echocardiography and Doppler examination was performed in all cases. Inclusion criteria were as follows: (a) fetuses without malformations and cases with neural tube defects, cerebral or skeletal malformations or aneuploidies without associated malformations, since previous analyses had revealed normal concentrations of nt-proBNP in fetal blood samples of affected cases [21]; (b) estimated fetal weight (EFW) appropriate for gestational age (AGA); (c) Doppler indices of the umbilical and middle cerebral artery within normal range; (c) qualitative echocardiography without any sign of myocardial dysfunction. Fetuses with any other type of malformation, infection, or monochorionic twin pregnancies were excluded. Neonatal notes were reviewed in all cases that ended in a life birth.

Maternal exclusion criteria were the presence of cardiac or renal diseases. In a subset of patients, maternal and fetal blood samples were collected for analysis of hemoglobin and nt-proBNP concentrations.

### 2.2. Ultrasound, Doppler examination and echocardiography

A detailed description of the fetal assessment, including ultrasound technique, Doppler studies and echocardiography has been published [17]. All examinations followed a standardized protocol and were performed with high-resolution equipment by specialists in fetal diagnostic imaging.

### 2.3. Sample analysis

Nt-proBNP was analyzed with a commercially available chemiluminescence immunoassay on a Dimension Vista 1500, and amniotic fluid total protein concentration (AF-TP) by photometry on a Dimension RxL Max System (Siemens Healthcare Diagnostics, Eschborn, Germany). For nt-proBNP, inter- and intra-assay coefficients of variation were 3.5% and 2.3%, respectively. All samples were processed within two hours after retrieval.

### 2.4. Statistical analysis

Correlation analysis was performed using Pearson's coefficient. To adjust for a potential dilutional effect, the AF-nt-proBNP/AF-TP ratio was analyzed. Reference intervals for the ln(AF-nt-proBNP/AF-TP) ratio were constructed according to Royston and Wright (1998) [22]. Fetal hemoglobin values were transformed into Multiples of the Median (MoM) [23].

## Results and Discussion

### 3.1. Results

**Figure 1:**
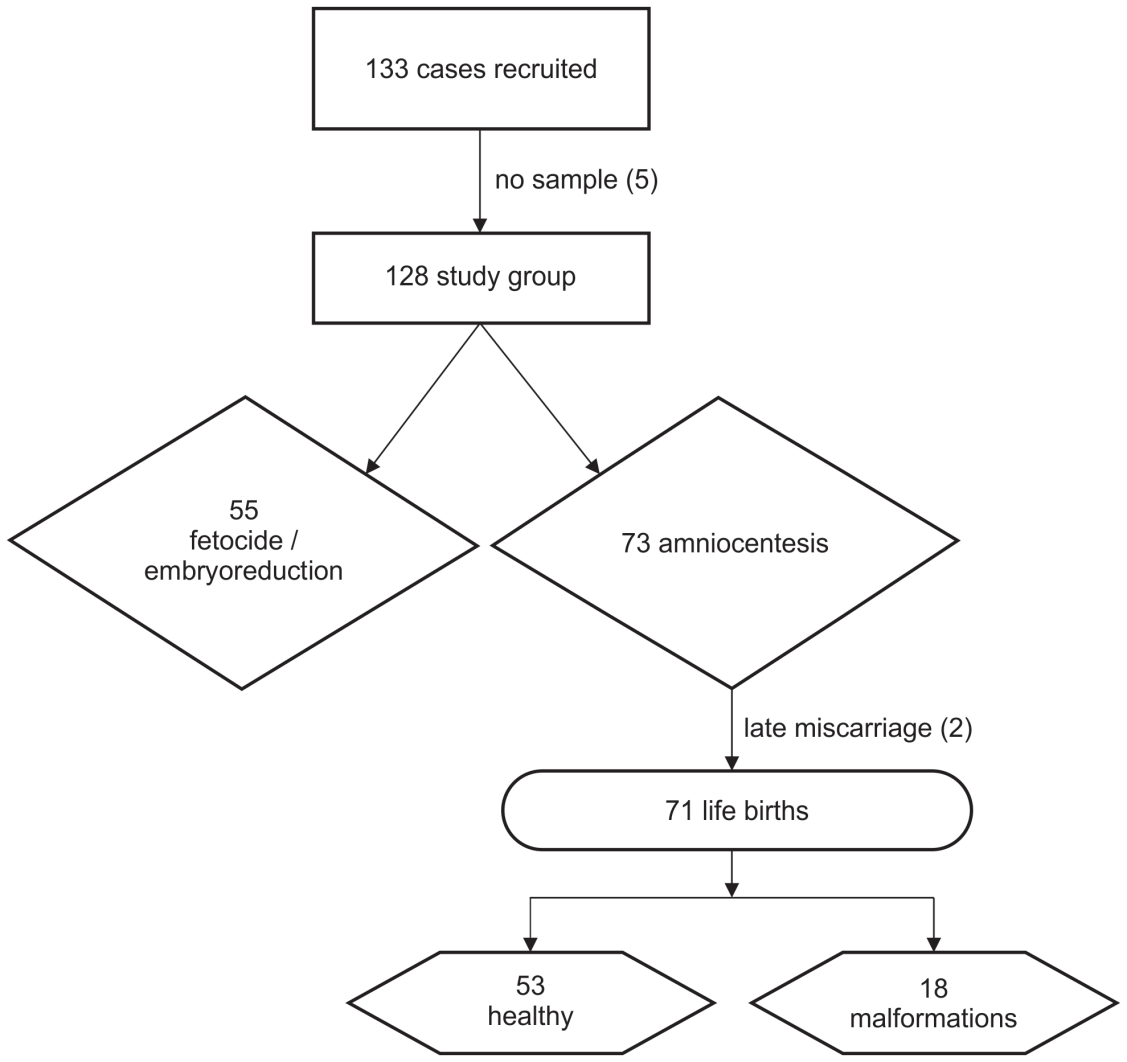
Study flow diagram.

**Figure 2:**
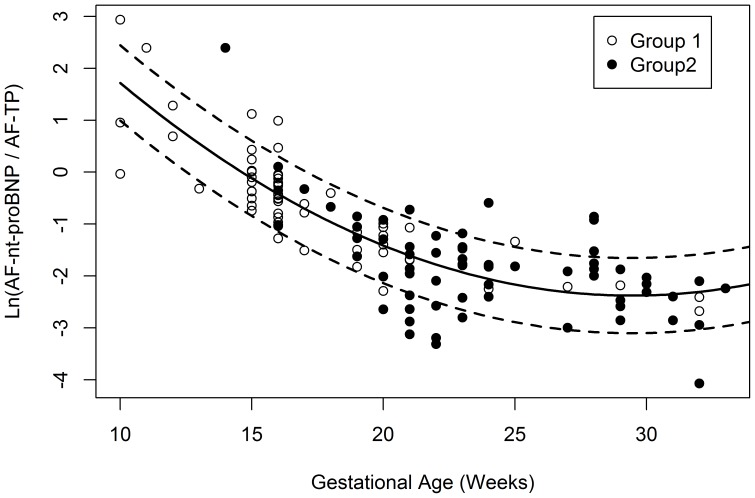
Curves for the mean and the 5% and 95% reference interval of ln(AF-nt-proBNP/AF-TP) between 10 and 34 weeks of gestation. Group 1, cases without malformations (n = 55); Group 2, cases with malformations (n = 73).

In total, 133 samples were collected. As AF-TP was not available in five cases, reference intervals were constructed with 128 samples. Fig. 1 illustrates the flow of the cases through the study. Clinical and obstetric details of participating women are listed in Table 1. Plasma nt-proBNP concentrations, determined in a subset of women, were within normal range. Variables with potential influence on maternal nt-proBNP concentration (hemoglobin, body mass index, age) were analyzed. No correlation was found between any of these parameters and nt-proBNP levels in maternal blood, fetal blood or amniotic fluid. Fetal details are listed in Table 2. Screening or diagnostic AC was the indication for 56.2% of cases; embryoreduction or fetocide was the indication for the others. For life births (57.4%), information on the outcome was retrieved from neonatal reports; prenatal diagnosis was confirmed in all cases. Fetal hemoglobin and plasma nt-proBNP concentrations were determined in 31 cases (24, respectively). A good correlation was found between nt-proBNP concentrations in amniotic fluid and fetal blood (r = 0.486; p<0.01); no correlation was present between nt-proBNP levels in fetal and maternal blood (maternal blood and amniotic fluid, respectively). An association of the analytes with GA was found (AF-nt-proBNP: r = −0.749; p<0.001; AF-TP: r = 0.175; p<0.05). Curves for the mean and 90% confidence limits of the logarithm of the AF-nt-proBNP/AF-TP ratio are depicted in Fig. 2. The equation for the curve is: y = 0.0109 GA^2^ – 0.630 GA +7.021. The variation around the curve was found to be constant (sd = 0.441); 5% and 95% percentile curves are calculated as mean +/− 0.441 * 1.645, the 95% quantile of the standardized normal distribution.

**Table 1:** Obstetric data (n = 128).

maternal age, mean (range)	33.7 (18–46)
**Parity, n (%)**	
**0**	41 (32.0)
**1**–**2**	77 (60.2)
**3**–**5**	10 (7.9)
**gestational age, mean (range)**	20.6 (10–33)
**BMI, mean (range)**	25.8 (17.9–41.5)
**hemoglobin, median (IQR)** *	12.0 (1.2)
**maternal plasma nt-proBNP, median (IQR)^§^**	54 (42)
**indication, n (%)**	
**diagnostic amniocentesis**	73 (57.0)
**embryoreduction/fetocide**	55 (43.0)

*available for 31 women; ^§^available for 24 women.

**Table 2:** Fetal data, including outcome (n = 128).

gestational age, mean (range)	20.6 (10–33)	
**sex, n (%)**		
**male**	68 (53.1)	
**female**	58 (45.3)	
**not known**	2 (1.6)	
**MoM** * **hemoglobin (%)**	≥0.84 (100)	
**fetal plasma nt-proBNP, median (IQR)^§^**	1699 (1496)	
**diagnosis, n (%)**		life birth
**no abnormality detected**	55 (43.0)	53 (96.4)
**cerebral malformation**	25 (19.5)	4 (16.0)
**skeletal malformation**	17 (13.3)	6 (35.3)
**neural tube defect**	11 (8.6)	0 (0)
**embryoreduction**	7 (5.4)	0 (0)
**others**	13 (10.2)	8 (61.5)

*MoM, Multiples of the Median, available for 26 cases; ^§^available for 31 cases.

Statistical tests comparing group specific regression parameters for the mean curve did not reveal evidence for differences between group one and two (cases with and without malformations).

### 3.2. Discussion

We successfully constructed reference intervals for amniotic fluid nt-proBNP between 10 and 34 weeks of gestation. Both, AF-nt-proBNP concentration and AF-nt-proBNP/AF-TP ratio decrease during the observation period. This finding reflects concentrations of nt-proBNP in fetal blood, albeit for earlier stages of gestation [21].

Several studies analyzed AF-nt-proBNP in TTTS. Concentrations correlated with the degree of myocardial dysfunction or disease severity. Controls were either not available or of small sample size [24]–[26]. A further study reported on amniotic fluid and umbilical cord BNP at delivery in monochorionic twin pregnancies; higher levels were present in newborns with weight discordance>20% or myocardial dysfunction [27].

### 3.3. Amniotic fluid composition and origin

The multitude of compartments contributing to AF inflow and outflow, the hydrostatic and osmotic gradients, and the changes that occur during the course of pregnancy have hampered the understanding of AF composition and dynamics [28]. Four factors are thought to be major AF determinants. They include urine and lung fluid which contribute to, and swallowing which reduces AF volume. Intramembranous transport, defined as fluid and solutes exchange that occurs at the part of the amnion that covers the fetal surface of the placenta, is considered the fourth major AF determinant [29]. It allows direct exchange between AF and fetal blood. Since intramembranous transport is the only extrafetal pathway contributing to AF, it may be the major factor regulating AF volume [29], [30]. Fetal urine constituents may additionally contribute to AF volume regulation [31]. In contrast, transmembranous exchange, occurring across the fetal membranes between AF and maternal blood within the uterine wall, is considered of importance only in early stages of pregnancy, deceasing to 0.3% of the AF volume with advancing gestation [32].

With respect to the origin of AF-nt-proBNP our findings and observations by other groups allow the conclusion that maternal origin of AF-nt-proBNP is unlikely. Transplacental exchange between the maternal and fetal compartment has been excluded [33], [34], and transmembranous transport seems highly unlikely, given the steep concentration gradient between maternal and AF-nt-proBNP and the lack of correlation.

Likewise, nt-proBNP synthesis within the fetal membranes (amnion and/or chorion) and transfer into the amniotic fluid seems unlikely. Itoh et al. (1993) [35] investigated BNP-like immunoreactivity in amniotic fluid, amnion tissue and cultured amniotic cells. Although BNP-like immunoreactivity was present in the amniotic fluid of all trimesters, no immunoreactivity was detected in third-trimester amnion tissue; expression in cell culture was detected after 10 days. Carvajal et al. (2009) [36] examined the effect of BNP extracted from fetal membranes on myometrial contractility. They concluded that BNP synthesized in the amnion enters the maternal compartment and exerts a paracrine effect on the myometrium.

Taken together, our data support the hypothesis that AF-nt-proBNP is of fetal origin. Our study does not allow a conclusion as to which fetal compartment(s) contribute to AF-nt-proBNP. Nt-proBNP has been detected in adult and neonatal urine [37], [38], concentrations seem to depend on renal function [39]. The composition of fetal lung fluid is largely unknown; pulmonary capillaries may excrete nt-proBNP. The intramembranous transport system may as well contribute to AF-nt-proBNP levels, given its direct impact on AF volume and composition [30], [40]. For clinical purposes, details about synthesis and metabolism are of minor relevance since the usefulness of a biomarker in clinical practice does not depend on the knowledge of the underlying pathomechanism [41].

### 3.4. Limitations

Ideally, reference intervals should be constructed with datasets from healthy cases. Furthermore, a longitudinal rather than a cross-sectional study design should be applied. Ethical considerations, however, prohibit an intervention with inherent complications for pure research purposes. In late stages of pregnancy, indications for amniocentesis are limited. We incorporated samples from fetuses with conditions where blood concentrations had been confirmed to be within normal range, and assume our findings to be valid. A general limitation of nt-proBNP analysis is the assay-specificity which hampers comparisons of studies performed with different assays [42].

## Conclusions

Indications for prenatal invasive therapy are on the rise [7]–[9] and diagnosis, intervention timing and treatment evaluation to date are limited to ultrasound-based methods. The availability of a biomarker would be useful for various aspects of fetal medicine, e.g. case selection, intervention timing, prognosis establishment and treatment evaluation in intrauterine therapy, and timing of delivery in cases on surveillance. Our reference intervals provide a basis for further research on AF-nt-proBNP as biomarker in fetal medicine.

## Supporting Information

S1 Table **Dataset.**
(XLS)
